# Peri-osteosarcoma adipose computed tomography attenuation and volume for predicting chemotherapy response in pediatric patients

**DOI:** 10.1007/s12094-022-03068-3

**Published:** 2023-01-14

**Authors:** Dong Fu

**Affiliations:** Department of Pediatric Orthopedics, Children’s Hospital of Fudan University, National Children’s Medical Center, Shanghai, 201102 China

**Keywords:** Chemotherapy response, Computed tomography, Osteosarcoma, Peri-osteosarcoma fat attenuation index, MTX blood concentration

## Abstract

**Background:**

The chemosensitivity of osteosarcoma patients to MTX is closely related to prognosis. There is currently a lack of advance prediction methods for MTX sensitivity.

**Objective:**

We proposed novel peri-osteosarcoma fat parameters based on computed tomography (CT) to evaluate the chemotherapy response preoperatively and calculate the correlation between image characteristics and methotrexate (MTX) blood concentration and systemic inflammation.

**Materials and methods:**

Pediatric patients with osteosarcoma (OS) who were treated with high-dose MTX were retrospectively studied and grouped according to postoperative Huvos classification. Clinical data were collected and reviewed. Image characteristics including peri-osteosarcoma fat volume and fat attenuation index were measured using the threshold method based on CT images. Statistical significance, correlation and prediction performance were performed.

**Results:**

Eighteen patients (good response (GR) group/poor response (PR) group: 10/8) was enrolled. MTX peak value at 6 h differed significantly between the two groups which was significantly higher in GR group (745.1 μmol/L vs 529.0 μmol/L *p* = 0.001). Peri-osteosarcoma fat attenuation index was significantly lower in GR group compared with that in PR group (− 104.90 vs. − 97.19, *p* < 0.0001). MTX blood concentration at 6 h negatively correlated with peri-osteosarcoma fat attenuation index (*R* = − 0.519, *p* = 0.027). In addition, 6 h MTX blood concentration (OR 0.974; 95% CI 0.951–0.998, *p* = 0.037) and FAI (OR 2.108; 95% CI 1.047–4.243, *p* = 0.037) were, respectively, independently related to good response to chemotherapy. The prediction performance on chemotherapy response of peri-osteosarcoma fat attenuation index and 6 h MTX blood concentration were both good with the comparable area under the ROC curve (0.950, 95% CI 0.856–1.000 and 0.963, 95% CI 0.878–1.00).

**Conclusions:**

Peri-osteosarcoma fat parameters based on CT were associated with the chemotherapy response and the MTX blood concentration, but not with the systemic inflammation. Combined with the requirement of current clinical practice, peri-osteosarcoma fat parameters may have the potential to be valuable image characteristics for monitoring chemotherapy response in OS pediatric patients.

## Introduction

Osteosarcoma (OS), originating from mesenchymal tissue, is the most common primary malignant tumor of bone, mainly affecting children and adolescents. OS is mostly seen in the metaphysis of long bones with a yearly incidence of 5.6 cases per million in children under the age of 15 [1]. Approximately, 5% of patients have primary metastases, and another 80–90% will develop metastases after only surgical resection. Therefore, in addition to surgery, systemic preoperative chemotherapy is needed to control the tumor [2]. Compared with surgery alone, combined chemotherapy before and after surgery can significantly increase the long-term survival rate [3, 4].

High-dose methotrexate (HDMTX) was the first effective drug to be introduced into the treatment of patients with OS by Jaffe [5]. Though MTX has been used as the first-line chemotherapy of OS for decades, the exact conditions for precise use of this drug have not been determined. In particular, the pharmacokinetics of MTX has not been established, although it is generally considered that higher doses (8–12 g/m^2^) are superior to lower doses (6 g/m^2^). Findings from Delepine shed a highly significant correlation between systemic tumor control and a critical MTX serum level (6 h MTX blood concentration) greater than 1000 µmol/L [6]. However, persistent high blood concentrations should be avoided because a poor elimination rate of MTX is associated with several side effects such as severe myelosuppression, renal dysfunction, liver dysfunction, oral stomatitis [7]. Therefore, it is extremely important to balance MTX peak value (MPV) and delayed elimination of MTX and identify factors associated with MPV and DEM to provide safe and more effective chemotherapy. Here, we have the question if there existed a potential parameter that would predict a child’s sensitivity to MTX.

Obesity is an important factor in the prognosis of carcinoma. Clinically, with the same dose of MTX, we found that the MPV of obese children is much lower than that of non-obese children, while the incidence of DEM is much higher. Therefore, it is not surprising to hypothesize that body composition may be associated with the effect of chemotherapy. A variety of previous studies have assessed the impact of body mass index (BMI) on operative and oncologic outcomes in patients with sarcomas, with a particular focus on sarcomas of the upper and lower limb [7–10]. However, few studies have assessed the impact of BMI on chemotherapy effect among patients with OS. In addition, BMI is not a precise measure of body composition, and research is increasingly focused on developing novel radiomic parameters of adipose tissue such as peri-osteosarcoma fat volume (PFV) and fat volume ratio (FVR) in various anatomic compartments based on CT images.

Systemic inflammation is another important factor affecting the prognosis of carcinoma, which is also known to have an effect on the pharmacokinetics of drugs. The efficacy of chemotherapy is sometimes limited due to systemic inflammation [11]. Recently, the radiomic parameter of peri-coronary fat attenuation index (FAI) has emerged as a noninvasive parameter of coronary inflammation and has been used to predict cardiovascular risk based on CT [12, 13]. We hypothesized that FAI of the adipose tissue around the tumor would show similar changes in the peri-coronary adipose tissue. Thus, we aimed to investigate the association between FAI measured on multidetector CT and systemic inflammation, pharmacokinetics of MTX among patients with OS.

In this paper, the volume and CT attenuation value of peri-osteosarcoma adipose were calculated to compare the disparities between pediatric patients with different responses to chemotherapy and to explore the correlation between the characteristics of adipose around tumor and inflammation and pharmacokinetics.

## Materials and methods

### Subjects

Patients with OS who were treated with HDMTX at our hospital from April 2018 to August 2021 were enrolled in this retrospective study. A total of 18 participants, including 7 boys and 11 girls with an average age of 8.78 years (range from 4 to 13 years) were included for the analyses. Ethics approval for this study was approved by the Human Research Ethics Committee at our hospital (No. 2021–157) and informed written consent was obtained from guardians of all children.

### Clinical data collection

The medical records were reviewed, for extraction of the following information pre-chemotherapy: age, gender, tumor location, weight, height, BMI, body surface area (BSA), MTX dosage, MTX blood concentration (6 h, 24 h, 42 h, 48 h, and 72 h), neutrophil and lymphocyte blood concentration, and neutrophil to lymphocyte ratio (NLR). BSA was derived from body weight using the following formulas:

$$BSA \left({m}^{2}\right)=0.035\times m+0.01 \left(m<30 kg\right)$$

$$BSA \left({m}^{2}\right)=\left(m-30\right)\times 0.02+1.05 \left(m>30 kg\right)$$

where *m (kg)* represents the body weight. If the BMI exceeds 25, the weight needs to be adjusted as the following formula:

$$Weight\, \left(adjusted\right)=\frac{AW-SWOSA}{4}+SWOSA$$

where *AW* represents the actual weight and *SWOSA* represents the standard weight of the same age. The initial dose of methotrexate is set as 10 g per BSA (m^2^).

### Definition of good and poor response

Patients were divided into the good response (GR) group and the poor response (PR) group according to the chemotherapy effect on the basis of Huvos tumor necrosis rate of the postoperative pathological specimens. Huvos tumor necrosis was graded as grade I (necrosis rate 0–49%), grade II (necrosis rate 50–89%), grade III (necrosis rate 90–99%), and grade IV (necrosis rate 100%). Grade III, IV referred to GR group and Grade I, II referred to PR group [3].

### Image acquisition

CT and MRI were conducted by experienced senior radiologists before chemotherapy. CT was performed using a 64-row multidetector scanner (LightSpeed VCT, GE Medical Systems) with the following parameters: 0.625 mm in slice collimation, 1.25 mm slice thickness, 512 × 512 in-plane resolution matrix, 50–60 mA of X-ray tube current and 100–120 kV of KVP. MRI was performed using a 1.5-T system (Avanto, Siemens, Erlangen, Germany) with the following parameters: T2-weighted imaging (repetition time/echo time = 3300/78 ms, echo train length = 5, number of excitations = 2), 320 × 160 in-plane resolution matrix. CT and MRI were performed at an interval of up to 2 days. All image data were stored in DICOM format.

### Quantitative and qualitative measurements of adipose tissue

**Fig. 1:**
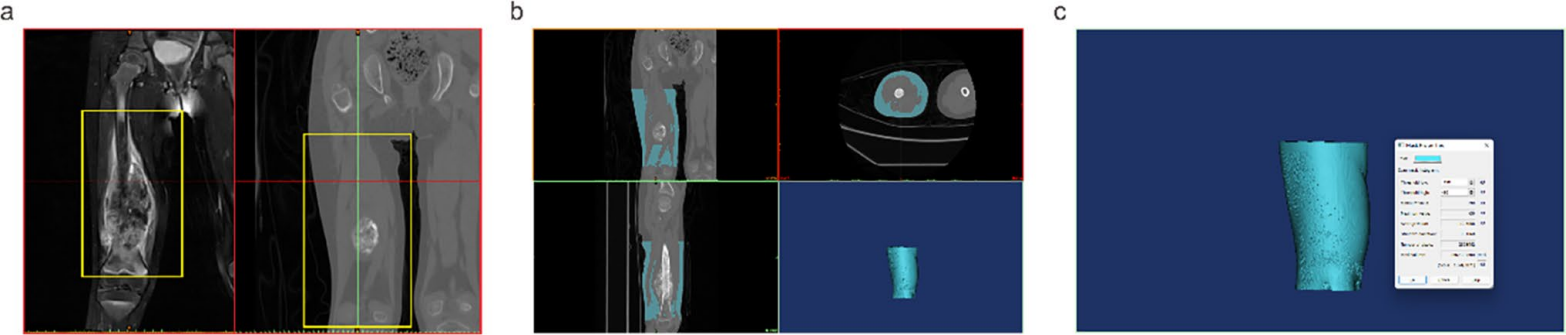
**a** Map of the OS region (yellow frame) on the MRI image to the CT image. **b** Establish the mask and the 3D anatomic model of the peri-osteosarcoma adipose. **c** The average CT attenuation and the total volume of the peri-osteosarcoma were displayed in the mask properties windows

The CT and MRI image data were imported into the Materialise’s interactive medical image control system 20.0 (MIMICS, Materialise, Belgium) to calculate the peri-osteosarcoma adipose characteristics by two experts in a blind fashion in the following steps: (1) mark the region of the OS on the MRI image and record the length of the OS, then map it to the CT image (Fig. 1a). (2) The threshold method was used to establish the mask and the 3D anatomic model of the peri-osteosarcoma adipose according to CT attenuation ranged from − 190 Hu to − 30 Hu, dotted mask was not considered as adipose and was removed (Fig. 1b). (3) Peri-osteosarcoma FAI defined as the average CT attenuation and the total volume of the peri-osteosarcoma was automatically calculated and displayed in the window of mask properties (Fig. 1c). (4) To standardize the adipose volume, the FVR was defined as the total volume of peri-osteosarcoma adipose divided by the length of the OS.

### Statistical analysis

General statistical analysis was performed using software IBM SPSS version 25 (IBM SPSS Statistics, Chicago, IL, USA) and GraphPad Prism version 9.3 (GraphPad Prism, California, USA). Continuous variables were presented as mean ± SD if normally distributed, and presented as median (range) if non-normally distributed. Categorical variables were presented as frequencies or percentages. The continuous variables were explored for normality by Shapiro–Wilk’s Test. Statistical significance was calculated using t-test, Welch test or Mann–Whitney *U* test according to the distribution of the variables. Intraclass correlation coefficient and Bland–Altman were used to analyze the interobserver agreement. Spearman rank correlation was calculated to determine the correlation between adipose characteristics and pharmacokinetics. Univariate and multivariate logistic regression analyses were adopted to determine adipose characteristics and pharmacokinetics related to good response to chemotherapy. The ROC curve of peri-osteosarcoma fat attenuation index and 6 h MTX blood concentration was drawn to assess prediction performance of adipose characteristics and pharmacokinetics. *p* value of < 0.05 was considered statistical significant.

## Results

### Patients’ baseline characteristics

A girl with MTX drug intoxication (the 6 h blood concentration and 24 h blood concentration were 2268 μmol/L and 224.64 μmol/L, respectively) was excluded for the subsequent analysis. Patients’ characteristics are summarized in Table 1. A total of 18 participants (age: 8.78 ± 2.37, boys/girls: 7/11) were enrolled in this study. OS was located at the left side in 9 patients (9/18) and at the right side in 9 patients (9/18). The GR group consisted of 10 patients (10/18) and the PR group of 8 patients (8/18). Age, BMI and BSA showed no significant difference between GR group and PR group.

**Table 1:** Demographics and patients’ characteristics (*N* = 18)

Age, year	
Mean ± SD	8.78 ± 2.37
Median	9.0
Minimum–maximum	4.0–13.0
Gender	
Boys	7 (7/18)
Girls	11 (11/18)
BMI (kg/m^2^)	17.96 ± 4.94
Weight (kg)	34.39 ± 15.23
Height (m)	1.36 ± 0.14
OS side	
Left	9 (9/18)
Right	9 (9/18)
BSA (m^2^)	1.09 ± 0.21
Groups	
Good response	10 (10/18)
Poor response	8 (10/18)

### Measurement reliability of CT-derived adipose characteristics

**Fig. 2:**
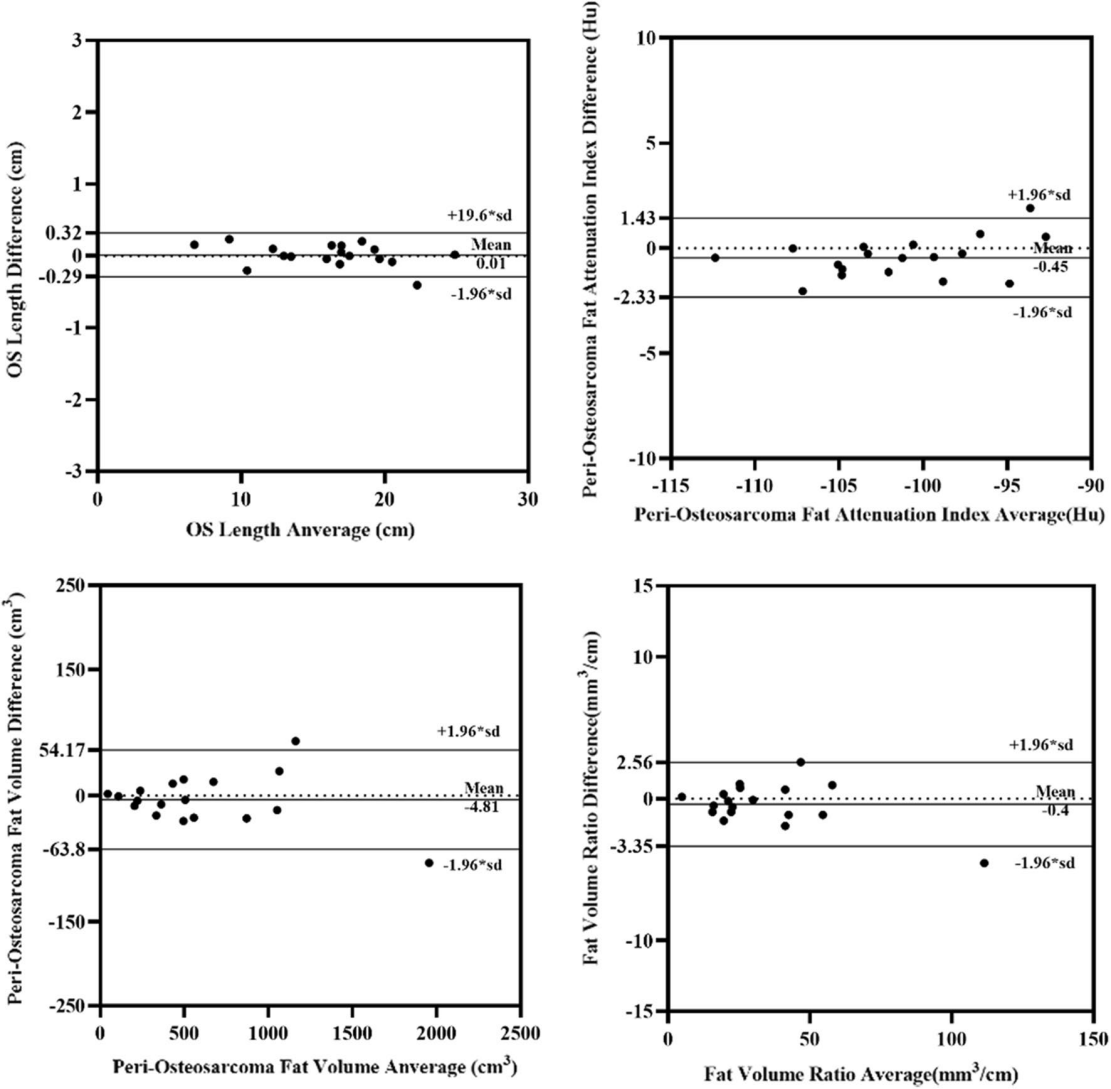
Differences between the pairs of measurements of CT-derived adipose characteristics

ICCs for two measurement results of CT-derived adipose characteristics are given in Table 2, respectively. Very good measurement agreement was seen in each CT-derived adipose characteristic (ICC = 0.999, 0.983, 0.998, 0.998, 0.397, *p* < 0.001, respectively) (Table 2). The difference between pairs of measurements of each CT-derived adipose characteristic was shown with the method of Bland–Altman analysis (Fig. 2). Measurement of CT-derived adipose characteristics was thought reliable.

**Table 2:** Intraclass correlation coefficient of CT-derived adipose characteristics

	Intraclass correlation	95% CI	*p* value
OS length (cm)	0.999	0.999–1.000	< 0.001
Peri-osteosarcoma fat attenuation index (Hu)	0.983	0.956–0.994	< 0.001
Peri-osteosarcoma fat volume (mm^3^)*	0.998	0.995–0.999	< 0.001
Fat volume ratio* (mm^3^/cm)	0.998	0.995–0.999	< 0.001

### Association of CT-derived adipose characteristics and patient-specific pharmacokinetics response

**Fig. 3:**
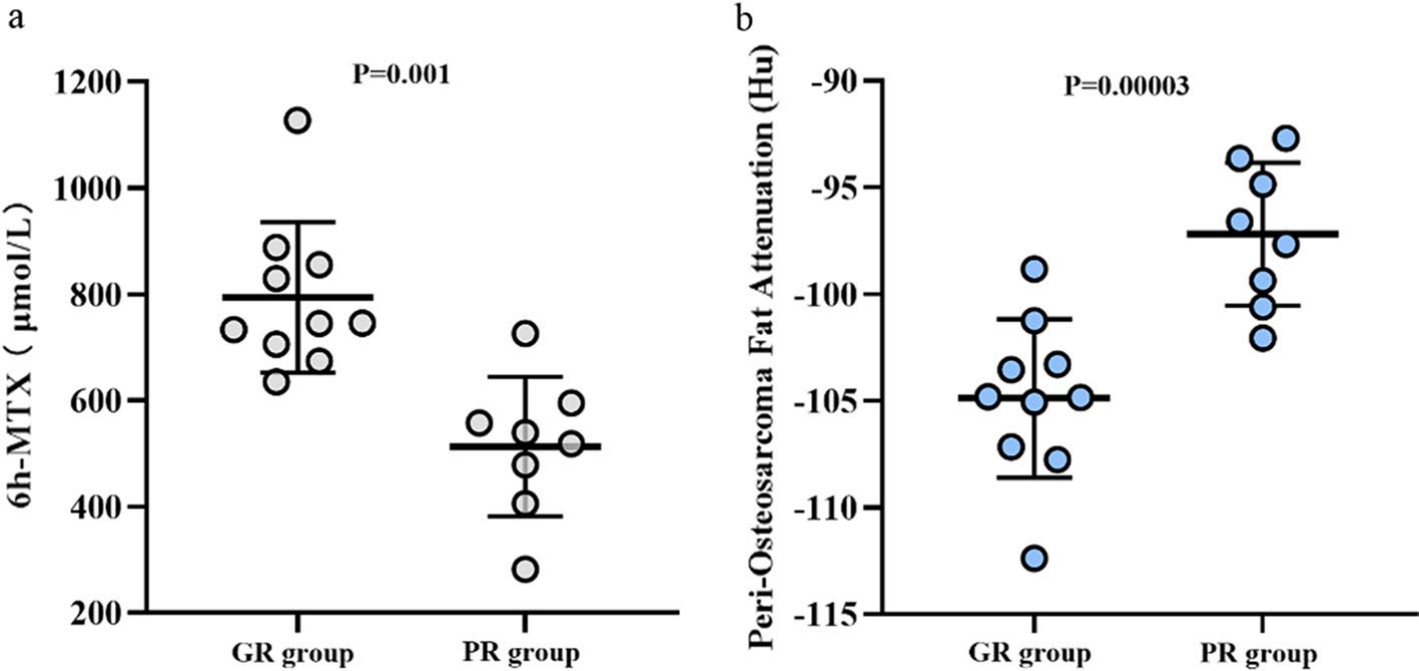
**a** Significant difference of 6 h MTX blood concentration between GR group and PR group. **b** Significant difference of peri-osteosarcoma FAI between GR group and PR group

MTX blood concentration at different time periods, inflammatory cells including neutrophil and lymphocyte, and the adipose characteristics are compared between GR group and PR group. 6 h MTX blood concentration differed significantly between the two groups, with significantly higher 6 h blood concentration in GR group than that in PR group (745.1 (697.6–863.5) vs. 529.0 (424.0–585.8), *p* = 0.001) (Table 3 and Fig. 3a), while 24 h, 42 h, 48 h, 72 h MTX blood concentration and NLR showed no significant differences between GR group and PR group.

**Table 3:** Comparison between GR group and PR group

	GR group (*N* = 10)	PR group (*N* = 8)	*p* value
Age	9.60 ± 2.5	7.75 ± 1.83	0.100^a^
BMI	17.00 ± 2.95	19.14 ± 6.71	0.425^b^
BSA*	1.14 (0.97–1.32)	1.03 (0.95–1.28)	0.515^c^
MTX blood concentration			
6 h (μmol/L)*	745.1 (697.6–863.5)	529.0 (424.0–585.8)	0.001^a^
24 h (μmol/L)*	7.54 (4.16–10.76)	10.40 (5.29–11.52)	0.315^c^
42 h (μmol/L)*	0.57 (0.46–0.76)	0.70 (0.49–1.06)	0.408^c^
48 h (μmol/L)*	0.34 (0.24–0.48)	0.48 (0.30–0.62)	0.237^c^
72 h (μmol/L)*	0.12 (0.05–0.18)	0.20 (0.10–0.44)	0.122^c^
Neutrophil (10^9^/L)	4.10 ± 0.85	5.28 ± 1.59	0.061^a^
Lymphocyte (10^9^/L)	2.77 ± 0.50	2.84 ± 0.63	0.797^a^
NLR	1.53 ± 0.47	2.11 ± 0.72	0.058^a^
OS length (cm)	14.31 ± 4.63	18.44 ± 3.73	0.058^a^
Peri-osteosarcoma fat attenuation index (Hu)	− 104.90 ± 3.71	− 97.19 ± 3.35	< 0.001^a^
Peri-osteosarcoma fat volume (mm^3^)*	433.19 (192.01–915.40)	494.76 (357.20–1039.44)	0.515^c^
Fat volume ratio* (mm^3^/cm)	26.28 (18.64–45.45)	25.30 (20.24–45.34)	0.829^c^

*Median

^a^*t* test for normal distribution and homoskedasticity

^b^Welch test for normal distribution and heteroskedasticity

^c^Mann–Whitney *U* test for non-normal distribution

In patients with good response to chemotherapy, peri-osteosarcoma FAI was significantly lower compared with that in patients with poor response (− 104.90 ± 3.71 Hu vs. − 97.19 ± 3.35 Hu, *p* < 0.001) (Table 3 and Fig. 3b). However, there was no evidence of a difference in fat volume and ratio was found between GR group and PR group.

### Correlation between adipose characteristics and pharmacokinetics

**Fig. 4:**
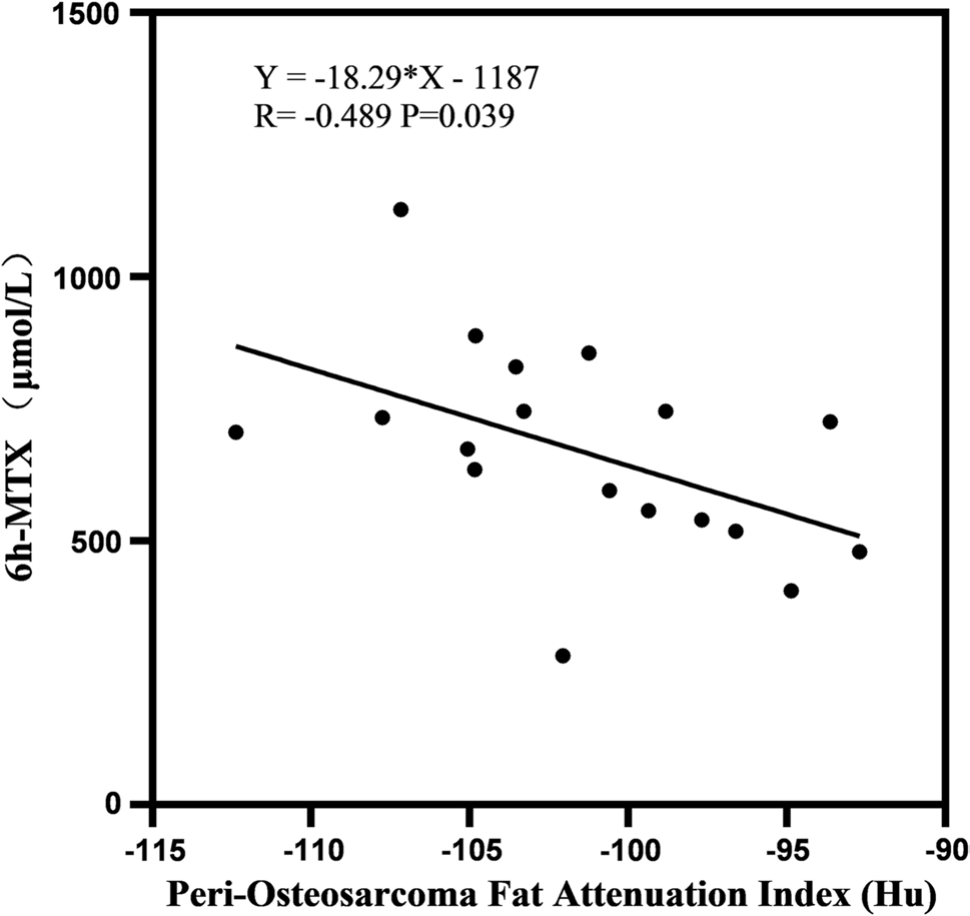
Correlation between PFV and 6 h MTX blood concentration

The correlation between fat-related parameters and MTX blood concentration at different time periods was determined using Spearman rank correlation (Table 4). It was shown that there was correlation between peri-osteosarcoma Fat attenuation index and 6 h MTX blood concentration (R = − 0.519, *p* = 0.027) (Table 4 and Fig. 4) rather than 24 h, 42 h, 48 h, and 72 h MTX blood concentration. FVR and PFV were uncorrelated with MTX blood concentration at 24 h, 42 h, 48 h, and 72 h. In addition, neither of FVR and PFV was associated with NLR. (*R* = 0.422 and 0.399, *p* = 0.081 and 0.101, respectively) (Table 4).

**Table 4:** Correlation between fat-related parameters and MTX blood concentration

	MTX blood concentration					NLR
	6 h	24 h	42 h	48 h	72 h	
OS length (cm)						
*R*	− 0.410	0.088	0.064	0.254	0.103	0.271
*p* value	0.091	0.729	0.801	0.309	0.685	0.278
Peri-osteosarcoma fat attenuation index (Hu)						
*R*	**− 0.519**	0.133	0.043	0.170	0.345	0.245
*p* value	0.027*	0.598	0.864	0.499	0.161	0.328
Peri-osteosarcoma fat volume (cm^3^)						
R	− 0.092	0.311	0.352	0.447	0.435	0.422
*p* value	0.717	0.210	0.151	0.063	0.071	0.081
Fat volume ratio (cm^3^/cm)						
*R*	− 0.018	0.296	0.373	0.420	0.397	0.399
*p* value	0.945	0.233	0.127	0.082	0.103	0.101

The [bold] means there exists a statistical difference

*P value < 0.05, Spearman rank correlation for non-normal distribution

### The univariate and multivariate logistic regression analyses

Univariate and multivariate logistic regression analyses were used to determine adipose characteristics and pharmacokinetics related to good response to chemotherapy. According to univariate logistic regression, independent association was found for 6 h MTX blood concentration (OR 0.974; 95% CI 0.951–0.998, *p* = 0.037) and FAI (OR 2.108; 95% CI 1.047–4.243, *p* = 0.037) (Table 5). On contrast, no variable was significantly associated according to multivariate logistic regression.

**Table 5:** Univariate and multivariate logistic regression analyses of adipose characteristics and pharmacokinetic

	Univariate			Multivariate
	Odds ratio	95% CI	*p* value	*p* value
MTX blood concentration				
6 h (μmol/L)	**0.974**	**0.951–0.998**	0.037*	0.999
24 h (μmol/L)	1.051	0.917–1.204	0.478	–
42 h (μmol/L)	5.029	0.059–430.136	0.477	–
48 h (μmol/L)	22.557	0.032–15,955.3	0.352	–
Neutrophil (10^9^/L)	2.405	0.871–6.643	0.090	0.999
Lymphocyte (10^9^/L)	1.283	0.219–7.527	0.783	–
NLR	5.397	0.827–35.234	0.078	0.999
OS length (cm)	1.296	0.965–1.742	0.085	0.999
Peri-osteosarcoma fat attenuation index (Hu)	**2.108**	**1.047–4.243**	0.037*	0.999
Peri-osteosarcoma fat volume (mm^3^)	1.001	0.999–1.003	0.343	–
Fat volume ratio (mm^3^/cm)	1.014	0.972–1.057	0.516	–

The [bold] mean there exists a statistical difference

### Prediction performance of adipose characteristics and pharmacokinetics

**Fig. 5:**
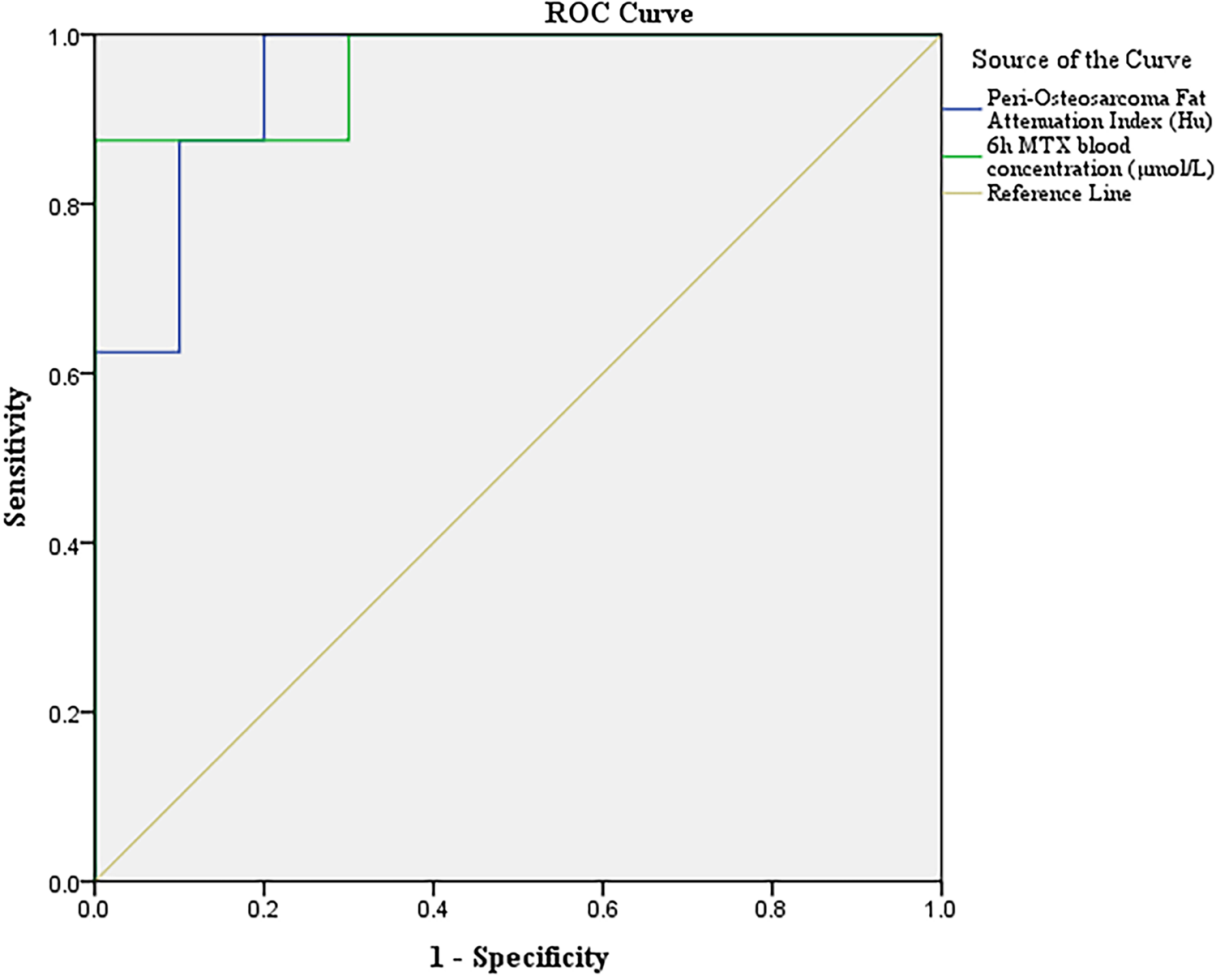
The ROC curve of peri-osteosarcoma fat attenuation index and 6 h MTX blood concentration

The ROC curve of peri-osteosarcoma fat attenuation index and 6 h MTX blood concentration is presented in Fig. 5. The area under the curve of peri-osteosarcoma fat attenuation index and 6 h MTX blood concentration were comparable (0.950, 95% CI 0.856–1.000 and 0.963, 95% CI 0.878–1.00) (Table 6). The accuracy, sensitivity, specificity, PPV, and NPV of peri-osteosarcoma fat attenuation Index and 6 h MTX blood concentration are, respectively, shown in Table 6.

**Table 6:** Prediction performance of adipose characteristics and pharmacokinetics

	AUC (95% CI)	Best cutoffs	Sensitivity	Specificity	PPV	NPV	Accuracy
Peri-osteosarcoma fat attenuation index	0.950 (0.856–1.000)	− 102.68	1.000	0.800	80%	100%	89%
6 h MTX blood concentration	0.963 (0.878–1.000)	615.175	0.875	1.000	100%	91%	94%

## Discussion

MAP chemotherapy for osteosarcoma is still the cornerstone of OS treatment. At present, the most commonly used method for scientific evaluation of drug sensitivity is the histological grading of tumor response to chemotherapy formulated by Huvos et al. Tumor necrosis rate assessment can only be performed after surgery with a significant lag, making early preoperative assessment difficult to achieve. In addition, the latest research found that some genes also have a certain effect of predicting chemotherapy efficacy, such as miR-138-5p, miR-1285-3p, and ERp29 genes [14–16]. However, there is still no accurate, convenient or inexpensive way to predict tumor chemotherapy sensitivity before treatment.

In this retrospective study, peri-osteosarcoma FAI, as a novel parameter of CT image, was found to be decreased in patients with good response chemotherapy. Consistent with previous findings [17], we also found the 6 h MTX blood concentration was with significantly higher in GR group. In addition, we found that peri-osteosarcoma FAI was significantly associated with the MTX blood concentration at 6 h. However, other adipose characteristics such as PFV and FVR were not associated with the MTX blood concentration which meant that MTX peak value and clearance was not affected by PFV and FVR.

Systemic inflammation in the tumor microenvironment is reported to have many tumor-promoting effects which include leading to the proliferation and survival of tumor cells and promoting angiogenesis and metastasis [18]. Application of anti-inflammatory interventions for patients with cancer has been suggested as important factors associated with the prognosis of carcinoma and may affect the sensitivity of chemotherapy [19]. Precious study has demonstrated that the combination of a high pretreatment platelet–lymphocyte ratio (> 116) and neutrophil count ≤ 4,030/μl was a useful inflammatory-based prognostic indicator for metastasis in patients with osteosarcoma [20]. But in this study, we did not find PFV and FVR were associated with NLR.

CT is a conventional imaging technique which is easily accessible from clinical data. Inflammation has been previously reported to be demonstrated by CT attenuation index, while fat volume as an indicator of obesity assessment was able to be obtained from CT images [21]. Therefore, peri-osteosarcoma FAI and the volume of peri-osteosarcoma fat derived from CT were selected as image markers in this study. To eliminate the interference of osteosarcoma size with the peri-osteosarcoma fat volume, the FVR defining as the total volume divided by the OS length was also calculated. The 6 h MTX blood concentration representative MPV was significantly higher in the GR group compared with the PR group, consistent with the results of previous studies [17, 22]. Our results also showed that peri-osteosarcoma FAI were significantly higher in the PR group compared with that in GR group as a reference. It was described that high FAI was associated with the degree of systemic inflammation. Inflammatory cytokines, such as tumor necrosis factor-α (TNF-α), interleukin-6 (IL-6), and interferon-γ (IFN-γ), trigger a local “cachexia” response in adipocytes in which the lipid phase of adipose tissue decreases and the aqueous phase increases. These series of changes can lead to higher FAI value around the lesion. FAI can not only be used to observe the existing inflammatory changes, but also to track the dynamic changes of inflammation to biological therapy, which is expected to be used for monitoring and evaluating the therapeutic effect of tumor [12].

There are some limitations to this study. First, potential doubts can arise about the power analysis of this limited sample, partially due to the low morbidity. A large multi-center prospective study is required for further demonstrating the validity of these parameters. Second, the CT and MRI images were not perfectly matched, causing the selection of the region of the tumor on CT to be subjective. Third, as the National Children’s Medical Center, our subjects were all from pediatric and were not necessarily applicable to adults.

## Conclusions

In conclusion, we found that peri-osteosarcoma fat parameters based on CT were associated with the chemotherapy response and the MTX blood concentration, which may have the potential to be a valuable image characteristic for predicting chemotherapy response preoperatively. It is worth mentioning that identifying such novel and simple markers may help distinguish high-risk patients who may need specific targeted therapy or immunotherapy in addition to conventional chemotherapy drugs.

## Data Availability

The datasets analyzed in this study are available from the corresponding author on reasonable request.
